# Roanoke’s Collective Public Health Activities

**DOI:** 10.13023/jah.0103.01

**Published:** 2019-09-27

**Authors:** Michael Lytton

**Keywords:** Appalachia, health disparities, life expectancy, racial segregation, economic segregation, Medically Underserved Areas, low income, Medicare

## Abstract

Roanoke is addressing problems that confront many small and medium sized cities in the U.S., especially disparities in health and life expectancy between neighborhoods. These disparities are often legacies of decades of racial and economic segregation, resulting in low-income or disinvested communities. Typically, such neighborhoods have fewer parks, higher vacancy rates and less stable affordable housing stock, inadequate public transit systems, too few clinics, too many fast food restaurants and insufficient access to high quality schools. In Roanoke these are the northwest and southeast quadrants, both federally designated Medically Underserved Areas, and characterized by a large proportion of the city’s low-income individuals and families who may be uninsured, underinsured and/or Medicaid recipients.

An article in this issue of the *Journal of Appalachian Health* by Bob Cowell Jr.,^1^ the City Manager of Roanoke, Virginia, complements his earlier piece in the Health Affairs Blog.^2^ Roanoke is addressing problems that confront many small and medium sized cities in the U.S., especially disparities in health and life expectancy between neighborhoods. These disparities are often legacies of decades of racial and economic segregation, resulting in low-income or disinvested communities. Typically, such neighborhoods have fewer parks, higher vacancy rates and less stable affordable housing stock, inadequate public transit systems, too few clinics, too many fast food restaurants and insufficient access to high quality schools. In Roanoke these are the northwest and southeast quadrants, both federally designated Medically Underserved Areas, and characterized by a large proportion of the city’s low-income individuals and families who may be uninsured, underinsured and/or Medicaid recipients.

Together, Bob’s two pieces describe collective public health activities in this mid-sized city that are remarkable in their depth, breadth and ambition. I have been researching the topic for over 5 years and am not aware of a more comprehensive program in the U.S. The constellation of actors working together includes city government, healthcare providers, schools, libraries, universities, colleges and a school of medicine, police and fire departments, free clinics, Habitat for Humanity, emergency shelters, local nonprofits, garden associations, countless volunteers, and more.

Cowell cites the 2012 Community Health Needs Assessment as the catalyst for a collective response, and the genesis of the “backbone” organization, Healthy Roanoke Valley, which brought together over 50 organizations and agencies. In his words, its mission was to “mobilize community resources to improve access to care, coordination of services, and promote a culture of wellness.”

The Roanoke Valley HOPE Initiative (RVHI) is illustrative. Offering treatment and recovery resources for individuals suffering from substance use disorders, RVHI is a collaborative effort among law enforcement, healthcare systems, treatment, recovery agencies, and safety-net organizations throughout the community. The coalition—today there are fourteen partner agencies—was formed in 2016 by the Roanoke Police Department, based on their acknowledgment that “we couldn’t arrest our way out of this crisis.” The HOPE Initiative has informed the community about the severity of the opioid (and now hepatitis C virus infection) epidemic, and successfully utilized peer recovery specialists and “Angel” volunteers.

Another critical player is the Center for Community Health Innovation (CCHI) at Roanoke College. Founded and directed by Dr. Liz Ackley, its primary functions include helping residents and partner organizations collaborate on equitable community development projects, and to identify challenges and develop innovative strategies to enhance healthy living in the Roanoke Valley. The Center is working with the City of Roanoke on its 2040 Comprehensive Plan, helping to craft policies that promote health and equity. The Center directs the Roanoke Valley Community Healthy Living Index, an annual assessment that monitors health outcomes, engagement in healthy behaviors, and barriers to healthy living among the City’s youth.

The Healthy Living Index is a unique experiential opportunity for students—service-driven data collection, analysis, and dissemination of materials that support local projects. “Through engagement with the Center, students gain real-world exposure to the processes involved in equity-driven, data-informed community development. This work substantiates the true value of a liberal arts education, as it requires students to develop the soft skills and technical skills necessary to work with partners in a variety of sectors, including residents, police officers, city officials, nonprofit leaders, and private developers,” says Ackley.

Roanoke is duly recognized for its progress. As Cowell points out, Roanoke has received seven All-America City Awards from the National Civic League and is the first city placed in its Hall of Fame. In addition, Roanoke was recently one of 12 cities chosen to join the National League of Cities pilot “Cities of Opportunity” initiative. The program, which receives support from the Robert Wood Johnson Foundation, focuses on three major factors that affect health: economic opportunity, housing, and city planning and design.

And I was pleased to learn recently that Roanoke College was the only academic institution among 23 grantees to receive financial and technical assistance awards through the Healthy Food Financing Initiative’s (HFFI) inaugural grants program.

So despite Cowell’s understandable impatience at the slow progress in poverty alleviation, achieving gains in the social/structural determinants of health—racism, poverty, transportation, housing, education, and diet—will take time and perseverance.

Antwyne Calloway, the Manager of the Healthy Roanoke Valley Pathways HUB says it best: We are finally having fruitful conversations about racism and segregation in our neighborhoods. People are listening to each other and not just reacting. Real progress is happening, but it takes time. We’ll get there.
